# Iodine

**Published:** 1862-02

**Authors:** 


IODINE.—We give the following extract from a Lecture on Iodine by S. B. Percy, M. D., Professor of Materia Medica and Therapeutics in New York Medical College. This is a subject interesting to the dentist; as this agent is coming into use as a topical application, its properties and influence should be well understood. We would suggest a close examination of this extract, as it contains, of course, the most recent observations on the subject.—ED. REG.

*Physiological Effects.*—Iodine is but seldom administered in a pure state, but is generally given in combination; but even if administered in a state of purity in medicinal doses, it no doubt quickly enters into organic or saline combinations, and in this way becomes milder and less irritant both in its local and general effects. Its local action is that of an irritant, whether applied to the mucous membrane or to the cuticle, and this effect may result whether applied in a solid, liquid, or aeriform state. It is at times very difficult to tell in what manner iodine affects the system, for it may be administered in small doses for a length of time without producing any noticeable alterations either in the functions of organs or on the secretions. There are many instances in which it is given for weeks, or even months, with no other perceptible effects than the amelioration or removal of the disease for which it is taken. In these small doses it generally improves the appetite, and this improvement continues until the system seems to be saturated; it then produces gastric disturbance. Even in large doses, its first effects are often a great improvement of appetite ; but if these doses are continued, there is anorexia, general symptoms of dyspepsia, unpleasant eructations, gastric irritability, frequently attended with colic and diarrhea ; the pulse becomes frequent and irritable, the tongue furred, the skin hot and dry, the respirations are more frequent, there is a peculiar sense of constriction and irritability about the throat, and much headache. If the medicine is discontinued, these effects soon pass over. As to its physiologlcal action on the secretions, its effects are very variable. Some persons notice a large increase in the quantity of urine, while others state that the seecretion is diminished, but that the flow of saliva is greatly increased. Again, it is said by some to largely increase the secretion of bile. It has been asserted that long-continued administration of iodine produces absorption of the mammæ and testicles, and there are probably a few cases reported where these glands have become atrophied and diminished ; but such cases are very rare, for Magendie, Lugol, and Pereira state that they have never met with such a result. By administration of iodine in full doses, there is an effect occasionally produced called *iodic intoxication*, or *iodism*, in which the nervous system is disordered, giving rise to headache, palpitation, ringing in the ears, dimness or disordered vision, irritability, fever, and wakefulness. Lugol, who administered iodine more largely than any one in his day, frequently produced these symptoms, not only by its internal use, but by means of indurretted baths. Manson also mentions similar cases. But these symptoms are the results of careless administration, and need not be, and I think are not frequently produced at present.

*Modus Operandi.*—Iodine is rapidly absorbed into the circulation, and can be detected in the secretions. From many experiments that have been performed, it appears that it is first to be detected in the saliva, then in the urine; sometimss it may be detected in the perspiration, but not as a rule, unless it has been taken for some time. Claude Bernard injected it into the jugular vein of a dog, and detected it immediately in the saliva, though it was not to be found in the urine until after the expiration of several hours. Schottin also found it in the saliva in a few minutes after administration, after some time in the urine, but in the perspiration it was not found until the fifth day. He gave half a drachm daily of iodide of potassium. Cantu has found it in the urine, sweat, saliva, milk, and blood. Meeting a few years ago with a person who had a salivary fistula, I tried some experiments to ascertain the rapidity with which iodine could be detected in the saliva and urine. I administered half a drachm of iodide of potassium in powder enveloped in a small piece of bibulous paper, which was put into the throat and immediately swallowed and followed by a gill of water. The bladder had been previously emptied and a small catheter introduced. The salivary secretion was immediately wiped away with a clean wet cloth, and one minute after the water was swallowed the saliva was collected and allowed to run into the spoon for two minutes. It was tested and gave evidence of the presence of the iodide. At the expiration of five minutes the urine was tested, but gave no traces; but in seven minutes after drinking the water, the urine ran off more freely, and all that passed from the seventh to the tenth minute was tested, and showed the presence of the iodide. The iodide could be detected in the saliva thirty hours after administration, though not a trace of it could be found in the urine. At another time I administered to him twenty drops of a saturated solution of tincture of iodine in gelatine capsules, followed as before by a gill of water. In three minutes it was found in the saliva, but it was twenty-two minutes before it was found in the urine. Pereira thinks that it produces its action upon the system by liquefaction of the blood. Billing states that it produces contraction of the capillary vessels, and others attribute its effects to direct stimulation (or rather increased action) of the absorbent system. There is but little doubt that all of these effects are produced, and the gentlemen who have advanced these separate theories have not adopted the usual custom and overstepped the mark, but have fallen short of it. In adopting the classification of Headland, and placing iodine in the third order of the second division of hæmatic medicines, we have already proved to some extent that he has correctly placed it under the division catalytics. We have given proof that it is absorbed into the blood through the coats of the stomach and intestines, that it enters into the portal circulation with great rapidity, and is found in a short time in several of the secretions, and also in the excretions; thus fulfilling the action of this class of remedies, by first entering into the circulating fluid, and counteracting a morbid material or process, and then passing out of the body. We have also given proof by the experiments performed with tincture of iodine, that it has undergone a change in the system, and has entered into new combinations, probably both organic and chemical. Independent of its catalytic effects, it might to a certain extent be placed under the division of restorative hæmatics, for we find it so universally diffused in nature that it must to some extent be one of the constituents of the system. We have proof, then, that before it produces its peculiar action on the system, it combines with organic substances, and is absorbed into the circulation; we have also proof, by more than one of its effects, that it hastens and in creases the metamorphosis of tissue, and by this means removes from the body the morbid materials which gave rise to the disease.

We see also in most instances a perceptible increase in one or other of the excretions, though in this respect it is not always the same. To prove that it hastens and increases the metamorphosis of tissue, let us watch its effects in both small and large doses. We find some morbid material or process in the system which produces a state of ill health; it is foreign to our purpose to inquire whether this material has been introduced into the system from ingesta, or is owing to a deficient power in certain organs to carry off the disintegrated and no longer needed substances. If in this state small doses of iodine are administered the only noticeable effects that it produces is an increased appetite, an increase in the specific gravity of the urine, and an absorption and removal of the materies morbi, with an increase in weight and renewal of health. But instead of giving it in small doses, let us see what are its effects when we administer it in large ones; after the first little increase of appetite caused by its stimulating effects, there is prostration and irritability, with loss of appetite. Absorption of the morbid material also takes place, the urine increases in specific gravity, and emaciation and loss of weight are readily noticed. In both instances we have then an increase in amount and rapidity of the metamorphosis of tissue, and this certainly in the first place without any stimulant action, as we understand the action of stimulants. That it should improve the appetite, and increase the strength and weight, when given in small doses, is readily explained, for we well know that all means that increase a healthy metamorphosis of tissue call for a corresponding effort of the nutritive process. In the second instance, where large doses are given and the metamorphosis of tissue thereby increased, increase of appetite and weight are prevented by the irritant effects of the medicine on the digestive organs. That it acts by increasing the metamorphosis of tissue we see also by its constant local action. An enlarged gland is painted over externally with tincture of iodine, and under its application the tumor disappears. I know that it is asserted that this is owing to its stimulating or counter-irritating effect; but it is not so, for the tumor is not discussed by application of tincture of capsicum, aqua ammonia, or nitrate of silver.—*American Medical Times.*

